# Do external female genital measurements affect genital perception and sexual function and orgasm?

**DOI:** 10.4274/tjod.galenos.2020.89896

**Published:** 2020-10-02

**Authors:** Aşkı Ellibeş Kaya, Ozan Doğan, Murat Yassa, Alper Başbuğ, Canan Özcan, Eray Çalışkan

**Affiliations:** 1 Düzce University Hospital, Clinic of Obstetrics and Gynecology, Düzce, Turkey; 2 Private Clinic, İstanbul, Turkey; 3 University of Health Sciences Turkey, Şehit Prof. Dr. İlhan Varank Sancaktepe Training and Research Hospital, Clinic of Obstetrics and Gynecology, İstanbul, Turkey; 4 Okan University Hospital, Clinic of Obstetrics and Gynecology, İstanbul, Turkey

**Keywords:** Female external genitalia, genital measurements, sexual function, genital perception

## Abstract

**Objective:**

To provide baseline data for the anatomy of the external female genitalia and to investigate the correlation between those measurements and sexual function and genital perception.

**Materials and Methods:**

This prospective cohort study consisted of 208 healthy premenopausal women. The Female Sexual Function index (FSFI) and the Female Genital Self-image scale (FGSIS) questionnaires were administered. Participants were divided into two groups according to their female sexual dysfunction (FSD) status. External genital measurements and anterior and posterior vaginal length were measured.

**Results:**

The external female genital measurements were (cm, mean ± standard deviation): clitoral prepuce length 2.05±0.48; clitoral glans length 0.87±0.21; clitoral glans width 0.60±0.15; clitoris to urethra 2.24±0.55; anterior fornix depth 7.75±0.92; posterior fornix depth 9.25±0.75; labia minora width, right 2.12±0.86, left 2.20±0.96. A weak negative correlation was found between total FGSIS scores and clitoral prepuce length (p=0.01, r=-0.17), whereas a weak positive correlation was seen between total FGSIS scores and anterior-posterior vaginal lengths (p=0.04, r=0.13; p=0.02, r=0.15, respectively). No statistically significant difference was found between the genital measurements of participants with FSD (n=82, 39.4%) and those without FSD (n=126, 60.6%), and the total FSFI scores and orgasm subdomain scores.

**Conclusion:**

The female genital measurements were found to be distributed over a wide range. Although the relationship between genital measurements and genital perception varied, no significant relationship was found between genital measurements and sexual functions or orgasm. These findings suggest that a more cautious approach should be taken towards genital surgeries for cosmetic purposes.

**PRECIS:** The female genital measurements were found to be distributed over a wide range and no significant relationship was found between genital measurements and sexual functions or orgasm.

## Introduction

External female genital measurements vary widely^(1,2,3,4,5)^. There are few reports in the literature regarding overall ‘normal’ female genital appearance or ‘normal’ dimensions, and exact positioning of the vagina, clitoris, and labia minora and majora^(1,5)^. There has been a significant increase in female genital cosmetic surgery rates over the years^(1,2,3,4,6)^. For the degree of increased female genital cosmetic surgery, the main motivator was found as improvement in genital appearance integrated with their aesthetic and sexual demands^(7)^.

It is known that body image and genital perception are related to sexual satisfaction^(8,9)^. Negative body image has been found to reduce sexual desire and arousability and was associated with fewer orgasms and less sexual satisfaction^(10)^. Among sexually active women, discomfort with the appearance of their genitals leads to anxiety and inhibitions during sexual activity^(11,12)^. A positive correlation has been determined between genital perception and sexual function^(13).^ However, it is not possible to evaluate sexual satisfaction and genital perception only with anatomic features due to the complexity and multifactorial structure of female sexuality^(14)^. It is important for the physician to recognize whether this discomfort is due to an anatomic reason or the perception of a defect. The media can negatively influence the genital perception of whom external genitalia are even in normal ranges^(6)^. In patients demanding an esthetic procedure in order to increase sexual satisfaction, an attempt should be made to correct genital perception before performing the surgery^(11)^.

Due to limited data on the distribution of genital measurements, this study aimed to provide baseline data for healthy, reproductive-age female external genital anatomic measurements and to determine the relationship between these measurements and genital perception and sexual function.

## Materials and Methods

Our prospective cohort study consisted of 208 healthy female participants. Our study included premenopausal patients aged over 18 years who were sexually active, and were seen in a medical faculty hospital polyclinic for routine gynecologic examinations between October 2017 and February 2018. The measurements and questionnaires were administered to healthy female participants who reported no known illnesses.

Exclusion criteria included postmenopausal and pregnant patients; those with previous vaginal and/or perineal, gynecological, or aesthetic surgical interventions; patients with stage >2 pelvic organ prolapse; urinary incontinence; menstrual irregularities; gynecological cancer; Polycystic Ovary syndrome (PCOS); patients using oral contraceptives or antidepressants; and those with intrauterine devices. Participants were taken to a quiet room, demographic data was recorded, and validated Turkish versions of the Female Sexual Function index (FSFI)^(15)^ and the Female Genital Self-image scale (FGSIS)^(16)^ were administered under the supervision of a physician.

The FSFI is a brief instrument consisting of 19 questions for the assessment of sexual function. Questions are scored for the domains of libido, arousal, lubrication, orgasm, satisfaction, and pain^(17)^. Female sexual dysfunction (FSD) was defined as a total score of 26 or less from a maximum possible score of 36^(18)^. The participants were divided into two groups according to their FSD status. The FGSIS is a seven-question survey that reveals female genital perception^(19)^. Genital perception is considered to be higher as the total score increases, with a maximum possible score of 28. The Beck Depression inventory was administered to patients who met the inclusion criteria and patients with a score of 17 and above were excluded from the study.

Subsequently, the participants were taken to the examination room and genital measurements were taken while in the lithotomy position. External genital measurements were made using a digital stainless-steel Vernier caliper, which can measure to 1/10 mm, and vaginal measurements were made using a hysterometer. The calipers were sterilized using ethylene oxide or were used by passing them through disposable bag gloves. The clitoral glans was measured by pulling back the prepuce. Labia minora and majora length and width were measured bilaterally. All measurements were made by two gynecologists who each had at least 10 years’ experience.

**Figure 1:**
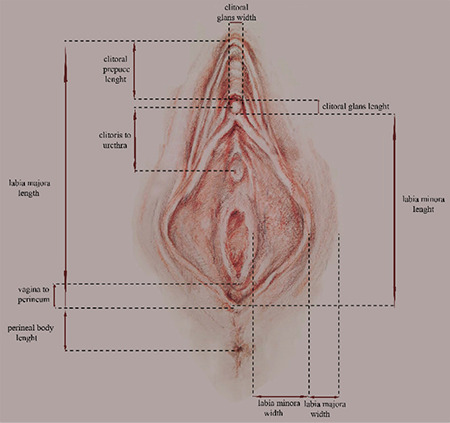
A schematic drawing of a part of the female external genitalia measurements. Labia minora width: The length from the base of the labium minora to the widest lateral prominence. Labia minora length: Longest craniocaudal length of the labium minora Clitoral glans width: Transverse diameter of the clitoral glans Clitoral glans length: Longest craniocaudal length of the clitoral glans Clitoral prepuce length: Length of the skin fold on the clitoris Clitoris to urethra: From mid-clitoral glans to mid-urethra Perineal body length: The length from the posterior fourchette to mid-anal orifice Vagina to perineum: From the point at the perineum where the labia minora begin to the hymen at 6 o’clock Labia majora length: Longest craniocaudal length of labium majus Labia majora width: Transvers length of labium majus

The template in Figure 1 was used for the genital measurements, which were taken according to the following definitions.

The primary outcome was the determination of the measurements of the external female genitalia. The FSFI and FGSIS were administered to the participants in order to understand the degree to which the results were clinically relevant. The secondary outcome was the determination of the relationship of the measurements to sexual function and genital perception and the relationship between genital measurements and age, body mass index (BMI), and parity.

### Ethical Approval

The institutional Ethics Committee approved the study (approval number: 2017/122), and written informed consent was obtained from all individual participants included in this study, which was conducted in accordance with the Helsinki Declaration.

### Statistical Analyses

Statistical analyses were performed using the Statistical Package for the Social Sciences for Windows 22.0 software (SPSS, Chicago, IL., USA). Descriptive statistics were calculated for subject demographics and dependent variables and were given as the mean, standard deviation, frequency and percentiles of 5%, 50% and 95%. Student’s t-test was used for the comparison of quantitative variables between groups. Correlations between continuous data were analyzed using the bivariate Pearson correlation coefficient. Relations between categorical data and genital parameters were evaluated using Pearson’s chi-square (χ^2^) test, depending on the type of variables. Statistical significance was defined as p<0.05.

## Results

The mean age of the participants in the study was 35.2±9.1 [mean ± standard deviation (SD); minimum =18, maximum =52] years, and the mean BMI was 25.1±4.6 (mean ± SD; minimum =16.3, maximum =41.5) kg/m^2^. Of the patients, 17.3% (n=36) were nulliparous and 82.7% (n=172) were multiparous. It was determined that 58.4% (n=101) of the multiparas had normal deliveries, 32% (n=55) delivered via caesarean section, and 9.3% (n=16) delivered both via caesarean and normally. Episiotomy was performed in 65.6% (n=66) of the 101 patients who delivered normally. Of the patients, 41.3% were smokers. When the genital measurements of the nulliparous and multiparous patients were compared, the anterior vaginal length was longer in multiparous patients (p=0.009) and the clitoris-urethra distance was shorter in multiparous patients (p=0.03). When comparing the types of delivery, the anterior and posterior vaginal lengths of the patients who had delivered normally were longer compared with those of patients who had undergone cesarean section (p=0.001, p=0.02, respectively). In patients who had undergone episiotomy, the anterior vaginal length was longer (p=0.008); there was no difference in posterior vaginal length (p=0.12).

The external female genital measurements were distributed over a wide range. The external female genital measurements detected were as follows: clitoral prepuce length 2.05±0.48; clitoral glans length 0.87±0.21; clitoral glans width 0.60±0.15; clitoris to urethra 2.24±0.55; anterior fornix depth 7.75±0.92; posterior fornix depth 9.25±0.75; labia minora width, right 2.12±0.86, left 2.20±0.96; labia minora length, right 3.60±1.17, left 3.79±1.26; labia majora width, right 3.02±0.59, left 2.98±0.63; labia majora length right 7.43±0.95, left 7.40±0.79 (cm, mean ± SD). The distribution of the genital measurements and the mean ± SD, range, and the 5, 50, and 95 percentile values are shown in Table 1.

According to the FSFI scores, a comparison of the patient group with FSD (n=82, 39.4%) and the group without FSD (n=126, 60.6%) is shown in Table 2. No statistically significant difference was found between the measurements of the two groups, and no statistically significant relationship was detected between genital measurements and total FSFI scores or the orgasm subdomain scores (Table 3).

Correlation analysis was performed between the genital measurements and the variable parameters of age, parity, and BMI (Table 3). There was a negative correlation between age and clitoris glans size and width and left labia minora width (p=0.02, r=-0.21; p=0.01, r=-0.01; p=0.02, r=-0.15, respectively). There was a weak positive correlation between parity and clitoral prepuce measurements (p=0.04, r=0.13). Moreover, a weak positive correlation between BMI and clitoris glans length was detected (p=0.01, r=0.17). No statistically significant relationship was found between BMI and total FSFI scores, FSFI orgasm sub-domain scores, or FGSIS scores (p=0.98, p=0.93, p=0.10, respectively).

A statistically significant weak negative correlation was detected between the total FGSIS scores and clitoral prepuce length, and there was a statistically significant weak positive correlation between the total FGSIS scores and anterior-posterior vaginal lengths (p=0.01, r=-0.17; p=0.04, r=0.13; p=0.02, r=0.15, respectively). As parity increased, the total FGSIS score decreased (p=0.001). There was a positive correlation between the total FSFI score and the total FGSIS score (p≤0.001, r*=*0.32).

## Discussion

A few studies have been conducted and they have shown that the range of female genital measurements can be quite extensive^(1,2,4,5)^. The current findings were consistent with the literature.

Vulvar morphology changes markedly with age^(5)^. Vagina size and labia minora width decrease with increasing age^(4,5)^. For this reason, postmenopausal patients were excluded from the study. Patients with PCOS were excluded because of the relationship between clitoral length and PCOS^(20)^.

The parameters of parity and BMI were also thought to influence genital measurements^(5)^. There are studies showing an increase in sexual dysfunction as BMI increases^(21,22)^. Although the desire and pain subdomains were unchanged with weight, a negative correlation was found between BMI and the orgasm, arousal, lubrication, and satisfaction subdomains^(22)^. As weight and abdominal circumference increased, the incidence of vaginal orgasm decreased and the masturbation rate increased^(21)^. This finding seems to support the psychological effect of the vaginal orgasm. In our study, changes in sexual function were not observed with increased BMI.

No difference was seen in vaginal size with an increase in parity^(1)^. In the current study, when we compared multiparous and nulliparous patients, it was determined that anterior vaginal length was longer in multiparous women. The vaginal depth of those who had delivered normally and of those who had undergone episiotomy was greater than in those who had undergone cesarean section. These results, due to the mechanism of birth, were not surprising.

The prevalence of FSD varies across the world, with a prevalence of 38.4% in the United States^(23)^. In our study, 39.4% of the patients were identified as having FSD, as defined in the literature.

Clitoral measurements were taken via magnetic resonance, and the clitoral measurements of anorgasmic subjects were found to be significantly smaller in a study by Oakley et al.^(24)^ Clitoral glans length measurements were between 1 and 2 cm and glans width was between 0.5 and 1 cm and no differences between clitoral dimensions according to age or weight were detected, whereas parity was found to increase the size of the clitoris in another study by Verkauf et al.^(25)^. In our study, clitoral measurements were consistent with the literature; however, although parity did not change the glans size, it was associated with an increase in the size of the prepuce.

In a study designed to directly examine sexual function with genital measurements using 32 samplings, no relation was found between genital measurements and sexual functions^(3)^. Our study, which used a wider sample, also found no significant difference between genital measurements and sexual function or orgasm.

Wallen and Lloyd^(26)^, in their study, analyzed row data of two separate older studies (Bonaparte 1933 and Landis 1940) and found different orgasm ratios below and above the clitoris-urethral meatus distance (CUMD) of 2.5 cm. A difference of 0.7 mm was found between the means of the CUMD (2.2 vs 2.9) in these two older studies. This difference was explained by the fact that in one study the measurement started from the middle of the glans, whereas the other started with the prepuce. We took our measurement from the middle of the glans, and our results were consistent with the literature. In our study, unlike in that study, no significant difference was found between the FSFI groups and the CUMD. Upon examination of the relationship between the FSFI orgasm subdomain and the genital perception scores, no significant difference was detected.

The FSFI orgasm subdomain was analyzed separately from the total score because it was thought that there could be a clinical relationship between the measurements and orgasm. However, no significant relationship was found between genital measurements, especially clitoral prepuce and labium minora length, and orgasm or sexual function. The very weak inverse relationship between genital perception scores and prepuce length can be explained by the influence of parity. With the increase in parity, an increase in the clitoral prepuce size and a decrease in genital perception were detected. This suggests that after clitoral hood reduction surgeries performed for the purpose of increasing sexual satisfaction, sexual outcomes should be compared with the status before the surgery.

Female genital esthetic operations were evaluated and sexual functions and their relationship with body image were investigated over a two-year period, in a prospective study^(11)^. The study found that, in the long term, these surgeries improved genital perception and sexual function. However, it was observed that the body-image perceptions, genital perceptions, and sexual functions of those who wanted surgery were lower than those of the control group^(11)^.

Many factors may affect genital perception^(27)^. It is also known that genital and body perceptions can be changed by exposure to visuals such as video and photographic images^(28)^. There was a positive correlation between FSFI and FGSIS, associating high genital perception with high sexual function^(11,13)^. Our study results are consistent with other studies that found a positive correlation between FSFI and FGSIS. For this reason, it is necessary to evaluate the pre-operation genital and body perceptions of women who desire esthetic surgery for reasons other than function.

### Study Limitations

The examination of female genital measurements and sexual functions along with genital perception created a powerful avenue of the study. The limitation of the study is that the study group reflected a part of society thought to be healthy. Multiparous patients were in the vast majority, and the lack of a categorical evaluation according to age and weight were other limitations. Another limitation was that the Body Dysmorphic Disorder scale was not administered to the patients. Future investigations dealing with the effects of surgical outcomes on sexual function and genital perception are recommended.

Considering the breadth of the distribution of the measurements, the fact that ‘normal’ female genital measurements can vary should be discussed with patients. For patients without functional impairment who want genital aesthetic surgery because of a desire to increase sexual satisfaction, an attempt should be made to first correct their genital perception. More research is needed on the subject of esthetic surgical procedures that change genital measurements and the contributions of this surgery to sexual function and genital perception.

## Conclusion

The wide range of genital measurements observed makes it difficult to draw the boundaries of ‘normal’ regarding female external genitalia. Genital cosmetic surgeries should be considered more cautiously because negative genital perceptions are open to verbal and visual influence and because there is no significant relationship between genital measurements and sexual functions.

## Figures and Tables

**Table 1:**
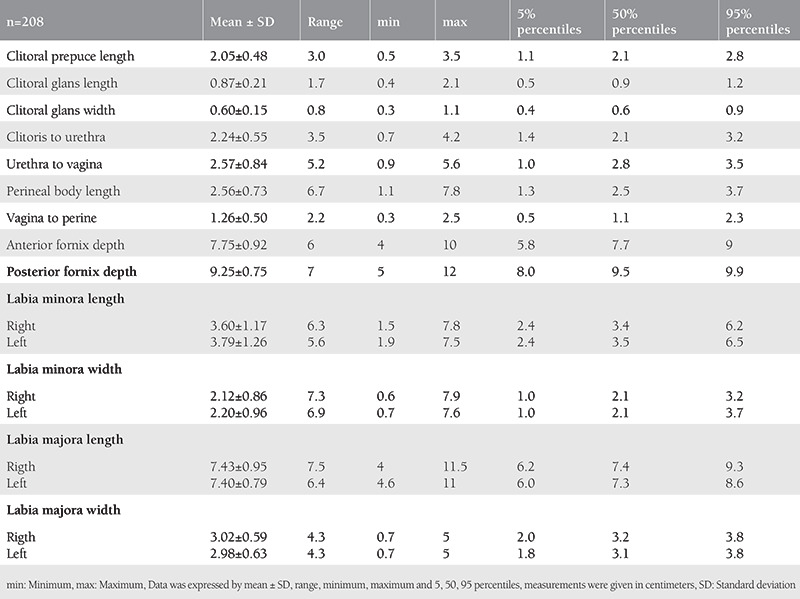
Distribution of genital measurements

**Table 2:**
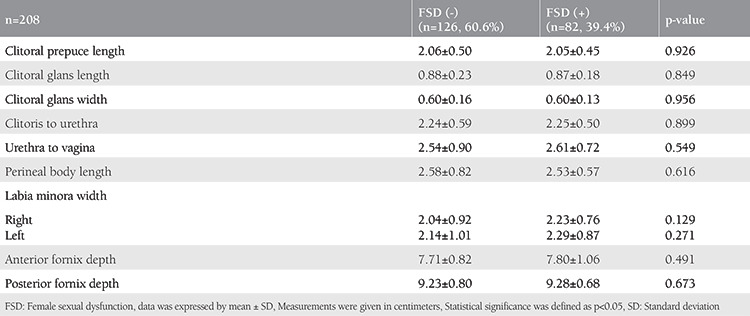
Comparison of genital measurements of patients with and without sexual dysfunction

**Table 3:**
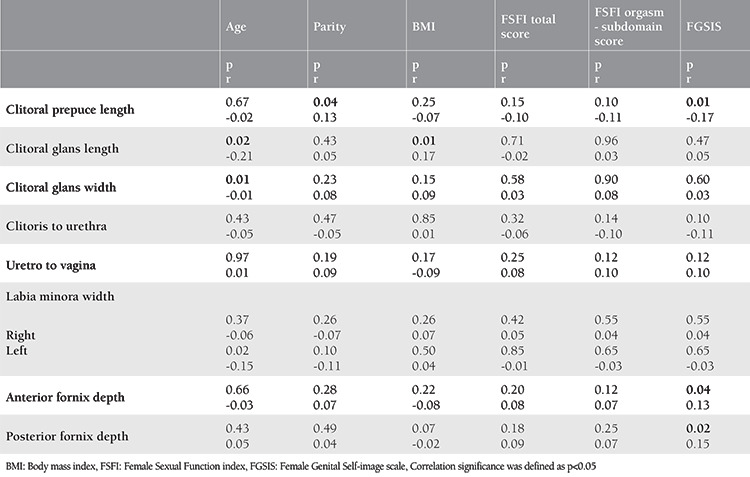
Correlation analysis of genital measurements and age, BMI, total FSFI scores, FSFI orgasm subdomain score and total FGSIS scores

## References

[1] Lloyd J, Crouch NS, Minto CL, Liao LM, Creighton SM (2005). Female genital appearance: “Normality” unfolds. BJOG An Int J Obstet Gynaecol.

[2] Cao Y, Li Q, Zhou C, Li F, Li S, Zhou Y (2015). Measurements of female genital appearance in Chinese adults seeking genital cosmetic surgery: a preliminary report from a gynecological center. Int Urogynecol J.

[3] Krissi H, Ben-Shitrit G, Aviram A, Weintraub AY, From A, Wiznitzer A, et al (2016). Anatomical diversity of the female external genitalia and its association to sexual function. Eur J Obstet Gynecol Reprod Biol.

[4] Basaran M, Kosif R, Bayar U, Civelek B (2008). Characteristics of external genitalia in pre- and postmenopausal women. Climacteric.

[5] Kreklau A, Vâz I, Oehme F, Strub F, Brechbühl R, Christmann C, et al (2018). Measurements of a ‘normal vulva’ in women aged 15–84: a cross-sectional prospective single-centre study. BJOG An Int J Obstet Gynaecol.

[6] Sharp G, Tiggemann M, Mattiske J (2016). Factors That Influence the Decision to Undergo Labiaplasty: Media, Relationships, and Psychological Well-Being. Aesthetic Surg J.

[7] Dogan O, Yassa M (2018). Major Motivators and Sociodemographic Features of Women Undergoing Labiaplasty. Aesthetic Surg J.

[8] Faith MS, Schare ML (1993). The role of body image in sexually avoidant behavior. Arch Sex Behav.

[9] Amos N, McCabe M (2016). Positive Perceptions of Genital Appearance and Feeling Sexually Attractive: Is It a Matter of Sexual Esteem?. Arch Sex Behav.

[10] Satinsky S, Reece M, Dennis B, Sanders S, Bardzell S (2012). An assessment of body appreciation and its relationship to sexual function in women. Body Image.

[11] Goodman MP, Placik OJ, Matlock DL, Simopoulos AF, Dalton TA, Veale D, et al (2016). Evaluation of body image and sexual satisfaction in women undergoing female genital plastic/cosmetic surgery. Aesthetic Surg J.

[12] La Rocque CL, Cioe J (2011). An Evaluation of the Relationship between Body Image and Sexual Avoidance. J Sex Res.

[13] Herbenick D, Schick V, Reece M, Sanders S, Dodge B, Fortenberry JD (2011). The Female Genital Self-Image Scale (FGSIS): Results from a Nationally Representative Probability Sample of Women in the United States. J Sex Med.

[14] Puppo V, Puppo G (2015). Anatomy of sex: Revision of the new anatomical terms used for the clitoris and the female orgasm by sexologists. Clin Anat.

[15] Oksuz E, Malhan S (2005). Reliability and validity of the Female Sexual Function Index in Turkish population. SENDROM.

[16] Ellibeş Kaya A, Yassa M, Dogan O, Basbug A, Pulatoğlu C, Caliskan E (2018). The Female Genital Self-Image Scale (FGSIS) - Cross cultural adaptation and validation of psychometric properties within a Turkish population. Int Urogynecol J.

[17] Rosen R, Brown C, Heiman J, Leib S (2000). The female sexual function index (FSFI): A multidimensional self-report instrument for the assessment of female sexual function. J Sex Marital Ther.

[18] Wiegel M, Meston C, Rosen R (2005). The Female Sexual Function Index (FSFI): Cross-validation and development of clinical cutoff scores. J Sex Marital Ther.

[19] Herbenick D, Reece M (2010). Development and validation of the female genital self-image scale. J Sex Med.

[20] Köşüş A, Kamalak Z, Köşüş N, Hizli D, Eser A (2016). Clitoral and labial sizes in women with PCOS. J Obstet Gynaecol.

[21] Costa RM, Brody S (2014). Orgasm and women’s waist circumference. Eur J Obstet Gynecol Reprod Biol.

[22] Esposito K, Ciotola M, Giugliano F, Bisogni C, Schisano B, Autorino R, et al (2007). Association of body weight with sexual function in women. Int J Impot Res.

[23] Buster JE (2013). Managing female sexual dysfunction. Fertil Steril.

[24] Oakley SH, Vaccaro CM, Crisp CC, Estanol MV, Fellner AN, Kleeman SD, et al (2014). Clitoral size and location in relation to sexual function using pelvic MRI. J Sex Med.

[25] Verkauf BS, Von Thron J, O’Brien WF (1992). Clitoral size in normal women. Obstet Gynecol.

[26] Wallen K, Lloyd EA (2011). Female Sexual Arousal: Genital Anatomy and Orgasm in Intercourse. Horm Behav.

[27] Ellibeş Kaya A, Doğan O (2018). Does educational level affect vulvar perception?. Turkiye Klin Jinekoloji Obstet.

[28] Moran C, Lee C (2013). What’s normal? Influencing women’s perceptions of normal genitalia: An experiment involving exposure to modified and nonmodified images. BJOG An Int J Obstet Gynaecol.

